# Structural and immunomodulatory differences among lactobacilli exopolysaccharides isolated from intestines of mice with experimentally induced inflammatory bowel disease

**DOI:** 10.1038/srep37613

**Published:** 2016-11-21

**Authors:** Sabina Górska, Corine Sandstrőm, Justyna Wojas-Turek, Joanna Rossowska, Elżbieta Pajtasz-Piasecka, Ewa Brzozowska, Andrzej Gamian

**Affiliations:** 1 L. Hirszfeld Institute of Immunology and Experimental Therapy, Polish Academy of Sciences, Weigla 12, 53-114 Wrocław, Poland; 2 Department of Chemistry and Biotechnology, Swedish University of Agricultural Sciences, P. O. Box 7015, SE-750 07, Uppsala, Sweden

## Abstract

Characteristic changes in the microbiota biostructure and a decreased tolerance to intestinal bacteria have been associated with inflammatory bowel disease (IBD). However, few studies have examined the constituents of the intestinal microbiota, including the surface molecules of the bacteria, in healthy and IBD subsets. Here, we compare the chemical structures and immunomodulatory properties of the exopolysaccharides (EPS) of lactobacilli isolated from mice with induced IBD (IBD “+”) versus those of healthy mice (IBD “−”). Classical structural analyses were performed using nuclear magnetic resonance spectroscopy and mass spectrometry. Immunomodulatory properties were assessed by stimulation of dendritic cells derived from mouse bone marrow or human peripheral mononuclear blood cells. Our results revealed that EPS produced by IBD “+” species are structurally different from those isolated from IBD “−”. Moreover, the structurally different EPS generate different immune responses by dendritic cells. We speculate that resident strains could, upon gut inflammation, switch to producing EPS with specific motifs that are absent from lactobacilli IBD “−”, and/or that bacteria with a particular EPS structure might inhabit the inflamed intestinal mucosa. This study may shed light on the role of EPS in IBD and help the development of a specific probiotic therapy for this disease.

Chronic inflammatory disorders of the digestive tract are known as inflammatory bowel disease (IBD). Ulcerative colitis and Crohn’s disease, which are the two major types of IBD, are associated with high morbidity and healthcare costs^1^. The incidence of IBD is increasing. This appears to be related to the “Western lifestyle,” with its high hygiene standards and rich diet, but the development and persistence of IBD involves other factors, some of which are complex. For example, disequilibration of the intestinal microbiota appears to play an important role. Using molecular techniques, a number of studies have identified changes in the composition of intestinal and fecal microbiota in patients with Crohn’s disease and ulcerative colitis, including reductions in mucosa-associated *Bifidobacterium* spp. or *Lactobacillus* spp., and increases in *Enterobacteriaceae*, including pathogenic *Escherichia coli*^2^. It is well known that gut microorganisms can modulate epithelial innate immunity, contribute to maintaining oral tolerance, protect against pathogens, and produce important metabolites (i.e., carbohydrates, vitamins, and short chain fatty acids)^3^. In recent years, researchers have sought to manipulate abnormal enteric microbiota to decrease pathogenic species and enhance the concentration and metabolic activity of beneficial species. This rational physiological and nontoxic approach has tremendous potential to confer therapeutic benefits, but it has not yet reached the point of clinical relevance. Moreover, very little is known of what constitutes a normal microbiota and what microorganisms are detrimental to immune homeostasis. Indeed, relatively few studies have examined the constituents of the intestinal microbiota, including the surface molecules of the bacteria, isolated from healthy and IBD subsets.

The ability of gut-resident bacteria to modulate immune responses appears to depend on their specific surface factors^4^. However, very little is known about the structure-function relationship of the molecules involved in bacteria-host crosstalk. A number of factors, including surface proteins, secreted proteins, teichoic acids and polysaccharides, have been shown to influence immune responses^5^. The polysaccharide antigens are highly diversified, varying in their component sugar monomers, linkages, chemical substitutions, branching and molecular mass. Recent studies have shown that these polymers have immunomodulatory properties and provide health benefits to the host^6^,^7^,^8^, and that they can be recognized by the dendritic cell (DC) and macrophage that are involved in bacterial recognition or antigen capture^9^.

In the early stages of bacterial recognition, DC play a pivotal role. DC are highly specialized antigen-presenting cells that have a unique capacity to establish and control the immune response. They recognize microorganisms, distinguish among them, and are able to induce different responses even to closely related organisms. This ability to respond appropriately to different microbial products is particularly important in the gastrointestinal tract, where diverse microbial flora are located in close proximity to DC^10^.

Here, we set out to compare the immunomodulatory properties of *Lactobacillus* strains isolated from mice with experimentally induced inflammatory bowel disease (IBD “+”, *L. johnsonii* 142, *L*. *animalis/murinus* 148, *L*. *animalis/murinus* 116, *L*. *reuteri* 115) and healthy mice (IBD “−”, *L. johnsonii* 151, *L*. *reuteri* 130), isolate exopolysaccharide (EPS) from these bacteria, and evaluate their structures and immunomodulatory effects. To study the structure-function relationship between the polysaccharides and their immunomodulatory activities, we examined the ability of select EPS to stimulate both mouse bone marrow-derived dendritic cells (BM-DC) and human mononuclear dendritic cells (Mo-DC) obtained from peripheral blood mononuclear cells (PBMC).

## Results

Exopolysaccharide fractions were isolated from each studied *Lactobacillus* strain, treated with DNase/RNase and protease to eliminate nucleic acids and proteins, respectively, and purified by ion-exchange chromatography on DEAE-Sephadex A-25. A typical chromatographic pattern comprised a neutral polysaccharide and fraction typical for a negatively charged polysaccharide. The neutral and charged fractions were purified by gel chromatography on a TSK HW-55S column. In total, we obtained nine exopolysaccharide samples: one from *L*. *johnsonii* 142 (E142, IBD “+”), one from *L*. *johnsonii* 151 (E151, IBD “−”), one from *L*. *reuteri* 130 (E130, IBD “−”), two from *L*. *animalis/murinus* 148 (E148, E148c, IBD “+”), two from *L*. *animalis/murinus* 116 (E116n, E116, IBD “+”), and two from *L*. *reuteri* 115 (E115n, E115, IBD “+”).

### Structural studies of exopolysaccharides isolated from *L. johnsonii* 142, *L*. *animalis/murinus* 148, *L*. *animalis/murinus* 116, *L*. *reuteri* 115 strains and from *L. johnsonii* 151, *L*. *reuteri* 130 strains

The GLC-MS analysis of the alditol acetates (sugar and methylation analysis) and the acetylated 2-butyl glycosides (determination of the absolute configuration) revealed that:

**E142** is composed of D-Glc and D-Gal in a molar ratio of 1:4. Methylation analysis revealed the presence of 5-substituted galactofuranose (1,4,5-tri-*O*-acetyl-2,3,6-tri-*O*-methylgalactitol-1-d), 3-substituted galactopyranose (1,3,5-tri-*O*-acetyl-2,4,6-tri-*O*-methylgalactitol-1-d), and 3-substituted glucopyranose (1,3,5-tri-*O*-acetyl-2,4,6-tri-*O*-methylglucitol-1-d) residues at a molar ratio of 1:3:1.

**E151** is composed of D-Glc and D-Gal in a molar ratio of 1:1.5. Methylation analysis revealed the presence of 3-substituted galactofuranose (resulting in 2,5,6-*O*-methyl-1,3,4-tri-*O*-acetyl-galactitol-1-d derivative), 2-substituted galactofuranose (3,5,6-tri-*O*-methyl-1,2,4-tri-*O*-acetyl-galactitol-1-d), 3-substituted glucopyranose (2,4,6-tri-*O*-methyl-1,3,5-tri-*O*-acetyl-glucitol-1-d), 6-substituted glucopyranose (2,3,4-tri-*O*-methyl-1,5,6-tri-*O*-acetyl-glucitol-1-d), and 6-substituted galactopyranose (2,3,4-tri-*O*-methyl-1,5,6-tri-*O*-acetyl-galactitol-1-d) residues in a 0.5:0.5:0.5:1.0:1.0 molar ratio.

**E130** is composed of D-Glc and D-Gal in a molar ratio of 1:1.3. Methylation analysis revealed the presence of 3-substituted galactofuranose (resulting in 2,5,6-*O*-methyl-1,3,4-tri-*O*-acetyl-galactitol-1-d derivative), 2-substituted galactofuranose (3,5,6-tri-*O*-methyl-1,2,4-tri-*O*-acetyl-galactitol-1-d), 3-substituted glucopyranose (2,4,6-tri-*O*-methyl-1,3,5-tri-*O*-acetyl-glucitol-1-d), 6-substituted glucopyranose (2,3,4-tri-*O*-methyl-1,5,6-tri-*O*-acetyl-glucitol-1-d), and 6-substituted galactopyranose (2,3,4-tri-*O*-methyl-1,5,6-tri-*O*-acetyl-galactitol-1-d) residues in a 0.5:0.4:0.6:0.9:1.0 molar ratio.

**E148** is composed of D-Glc, D-Gal, D-ManNAc and D-GalNAc in a molar ratio of 1.0:0.5:0.4:0.8. Methylation analysis revealed the presence of 4,6-substituted glucopyranose (2,3-di-*O*-methyl-1,4,5,6-tetra-*O*-acetyl-glucitol-1-d), 3,4-substituted galactopyranose or 3,5-substituted galactofuranose (1,4,5-tri-*O*-acetyl-2,3,6-tri-*O*-methyl-D-galactitol-1-*d*), 3-substituted galactosamine (1,3,5-tri-*O*-acetyl-2-amino-4,6-di-*O*-methyl-D-galactitol), 6-substituted mannosamine (1,5,6-tri-*O*-acetyl-2-amino-3,4-di-*O*-methyl-D-mannitol), and terminal glucopyranose (1,5-di-O-acetyl-2,3,4,6-tetra-O-methyl-d-glucitol) residues in a molar ratio of 0.5:0.5:1.0:0.5:1.0.

**E148c** is composed of D-Glc and D-Gal in a molar ratio of 1.0:1.2. Methylation analysis revealed the presence of 4-substituted glucopyranose (1,4,5-tri-*O*-acetyl-2,3,6-tri-*O*-methyl-D-gluctitol-1-*d*), 2-substituted glucopyranose (1,2,5-tri-*O*-acetyl-3,4,6-tri-*O*-methyl-D-glucitol-1-*d*), terminal galactofuranose (1,5-di-O-acetyl-2,3,4,6-tetra-O-methyl-d-galactitol), 6-substituted glucopyranose (1,4,6,-tri-*O*-acetyl-2,3,5-tri-*O*-methyl-D-galacitol-1-*d*), 3,6-disubstituted galactopyranose (1,3,4,6-tetra-*O*-acetyl-2,5-di-*O*-methyl-D-galactitol-1-*d*), and 3-substituted galactopyranose (1,3,5-tri-*O*-acetyl-2,4,6-tri-*O*-methyl-D-galactitol-1-*d*) residues in a molar ratio of 0.7:1.0:1.1:1.2:0.7:1.1.

**E116n** is composed of D-Glc, D-Gal, D-ManNAc, and D-GalNAc in a molar ratio of 1.0:0.5:0.5:1.0. Methylation analysis revealed the presence of 4,6-substituted glucopyranose (2,3-di-*O*-methyl-1,4,5,6-tetra-*O*-acetyl-glucitol-1-d), 3,4-substituted galactopyranose or 3,5-substituted galactofuranose (1,4,5-tri-*O*-acetyl-2,3,6-tri-*O*-methyl-D-galactitol-1-*d*), 3-substituted galactosamine (1,3,5-tri-*O*-acetyl-2-amino-4,6-di-*O*-methyl-D-galactitol), 6-substituted mannosamine (1,5,6-tri-*O*-acetyl-2-amino-3,4-di-*O*-methyl-D-mannitol), and non-substituted glucopyranose (1,5-di-O-acetyl-2,3,4,6-tetra-O-methyl-d-glucitol) residues in a molar ratio of 0.5:0.3:0.7:0.5:1.0.

**E116** is composed of D-Glc and D-Gal in a molar ratio of 1.0:1.0. Methylation analysis revealed the presence of 4-substituted glucopyranose (1,4,5-tri-*O*-acetyl-2,3,6-tri-*O*-methyl-D-gluctitol-1-*d*), 2-substituted glucopyranose (1,2,5-tri-*O*-acetyl-3,4,6-tri-*O*-methyl-D-glucitol-1-*d*), terminal galactofuranose (1,5-di-O-acetyl-2,3,4,6-tetra-O-methyl-d-galactitol), 6-substituted glucopyranose (1,4,6,-tri-*O*-acetyl-2,3,5-tri-*O*-methyl-D-galacitol-1-*d*), 3,6-disubstituted galactopyranose (1,3,4,6-tetra-*O*-acetyl-2,5-di-*O*-methyl-D-galactitol-1-*d*), and 3-substituted galactopyranose (1,3,5-tri-*O*-acetyl-2,4,6-tri-*O*-methyl-D-galactitol-1-*d*) residues in a molar ratio of 1.0:1.0:1.1:1.0:0.9:1.1.

**E115n** is composed of D-Glc and D-Gal in a molar ratio of 1:1.4. Methylation analysis revealed the presence of 3-substituted galactofuranose (resulting in 2,5,6-*O*-methyl-1,3,4-tri-*O*-acetyl-galactitol-1-d derivative), 2-substituted galactofuranose (3,5,6-tri-*O*-methyl-1,2,4-tri-*O*-acetyl-galactitol-1-d), 3-substituted glucopyranose (2,4,6-tri-*O*-methyl-1,3,5-tri-*O*-acetyl-glucitol-1-d), 6-substituted glucopyranose (2,3,4-tri-*O*-methyl-1,5,6-tri-*O*-acetyl-glucitol-1-d), and 6-substituted galactopyranose (2,3,4-tri-*O*-methyl-1,5,6-tri-*O*-acetyl-galactitol-1-d) residues in a 0.6:0.4:0.5:1.0:0.9 molar ratio.

**E115** is composed of D-Glc, D-Gal, and D-GlcNAc in a molar ratio of 1.0:1.5.1.1 Methylation analysis revealed the presence of 3-substituted glucopyranose (1,3,5-tri-*O*-acetyl-2,4,6-tri-*O*-methyl-D-glucitol-1-*d*), 3-substituted galactopyranose (1,3,5-tri-*O*-acetyl-2,4,6-tri-*O*-methyl-D-galactitol-1-*d*), 4-substituted galactopyranose or 5-substituted galactofuranose (1,4,5-tri-*O*-acetyl-2,3,6-tri-*O*-methyl-D-galactitol-1-*d*), 2,3-disubstituted glucopyranose (1,2,3,5-tetra-*O*-acetyl-3,6-di-*O*-methyl-D-glucitol-1-*d*), 6-substituted galactofuranose (1,5,6-tri-*O*-acetyl-2,3,4-tri-*O*-methyl-D-galactitol-1-*d*), 3-substituted glucosamine (1,3,5-tri-*O*-acetyl-2-amino-4,6-di-*O*-methyl-D-glucitol), and 6-substituted glucosamine (1,5,6-tri-*O*-acetyl-2-amino-3,4-di-*O*-methyl-D-glucitol) residues in a 1.0:0.9:1.0:1.0:0.9:1.0:1.0:0.8 molar ratio.

Our NMR analysis determined that E130 and E115n were structurally identical to E151^11^. The structure of E142 was exactly the same as one previously reported by our group^12^. The E148 and E116n structures were the same as previously reported for L900/3^6^ and L919/B^13^. E115 and E116 (E148c) exhibited uncommon and new chemical structures, and were thus subjected to detailed NMR analyses.

### NMR analysis of E115

**Figure 1:**
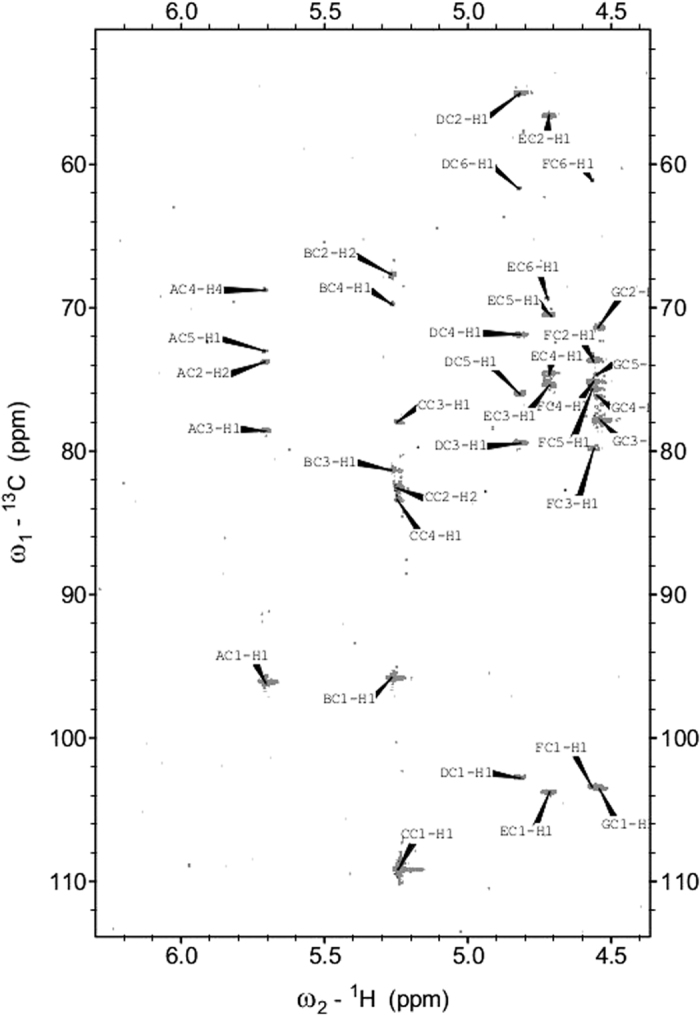
Selected portion of the ^1^H-^13^C HSQC-TOCSY spectrum of the E115 exopolysaccharide from *Lactobacillus reuteri* 115. Capital letters denote the sugar residues.

**Figure 2:**
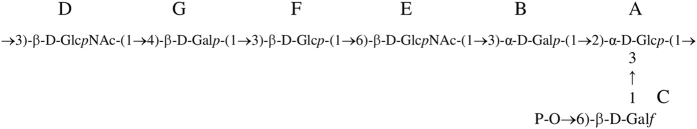
Structure of the E115 polysaccharide. Capital letters refer to sugar residues.

The one-dimensional ^1^H NMR spectrum obtained at 600 MHz for the E115 sample revealed seven anomeric proton signals, which were observed at 5.71, 5.27, 5.26, 4.81, 4.71, 4.56 and 4.54 ppm. In the HSQC spectrum, the proton signals were correlated with seven carbon resonances; these were observed at 96.1, 95.7, 108.8, 102.8, 103.8, 103.5, and 103.6 ppm, respectively. The HSQC spectrum also contained two signals (δ_H_ 1.93 and 1.95 ppm and δ_C_ at 22.1 and 22.3 ppm) that are characteristic of methyl groups from the acetyl group in an acetamido sugar. The ^31^P-NMR spectrum contained one signal for a phosphomonoester group at δp 0.53 ppm. The seven monosaccharides of E115 were designated as residues **A-G** in decreasing order of their proton chemical shift values. The ^1^H and ^13^C chemical shifts for all components of E115 were assigned from two-dimensional COSY, TOCSY, HSQC, and HSQC-TOCSY spectra (Fig. 1), as well as by comparison with ^1^H and ^13^C NMR data that were previously published^14^ and are reported in Table 1. The spin systems of the seven sugar residues were identified based on typical ^3^*J*_H_,_H_ coupling constant values estimated from the cross-peaks in the TOCSY spectra^15^. The TOCSY spectra showed cross-peaks of H1 with H2-H5 for residues **A**, **B**, **C**, **F**, and **G**, whereas we obtained cross-peaks of H1 with H2-H4 for **D** and **E.** Assignment was completed by the following correlations in the COSY spectrum: H5/H6 for **A, B, C, F** and **G**; and H4/H5 and H5/H6 for **D** and **E**. Our identification of the spin systems of the *N*-acetylamino sugars was confirmed by correlating protons at nitrogen-bearing carbons with the corresponding carbons at 54.94 ppm (C2 of **D**) and 56.42 ppm (C2 of **E**) in the HSQC experiment. Analyses of the TOCSY spectra and ^1^H and ^13^C chemical shifts allowed residues **B, C**, and **G** to be assigned as having the *galacto*-configuration (a large vicinal coupling constant between H-2 and H-3, and small vicinal coupling constants between H-3, H-4, and H-5) whereas residues **A, D, E, F** were assigned as having the *gluco*-configuration (large coupling constants for the all spin system). The four anomeric resonances of **D, E, F**, and **G** had large ^3^*J*_H1,H2_ coupling constants, indicating that they are β-linked pyranosidic residues. Residues **A** and **B** were identified as α-linked pyranosidic residues, and **C** as a β-linked furanosidic residue. The ^1^H and ^13^C chemical shifts allowed us to identify residues **A, B, C, D**, **E, F**, and **G** as glucose, galactose, galactose, two *N*-acetylglucosamines, glucose, and galactose, respectively. The sequence of the monosaccharide residues within the repeating unit of the E115 polysaccharide was obtained by assignment of the inter-residue connectivities observed in the 2D NOESY and HMBC spectra (Table 2). The position of substitution for the phosphate was revealed by ^1^H,^31^P-correlation experiments (HMQC and HSQC). The ^31^P resonance at *δ* 0.53 ppm showed connectivity to signals at *δ* 4.22 and 4.14 ppm (the H-6 of residue **C**). The final elucidated structure of the heptasaccharide repeating unit of the *L. reuteri* 115 polysaccharide, E115, is shown in Fig. 2.

### NMR analysis of E116 (E148c)

**Figure 3:**
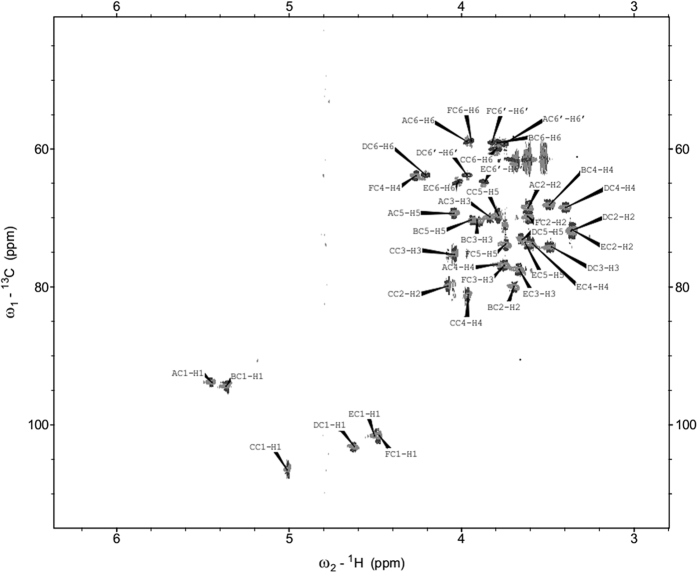
Selected portion of the ^1^H-^13^C HSQC spectrum of the E116 exopolysaccharide from *Lactobacillus animalis*/*murinus* 116. Capital letters refer to sugar residues.

**Figure 4:**
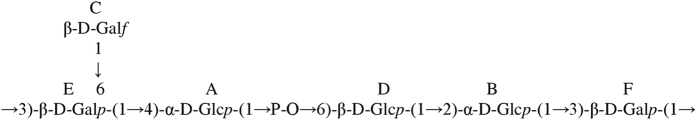
Structure of the E116 (E148c) polysaccharide. Capital letters refer to sugar residues.

The one-dimensional ^1^H NMR spectrum of E116 revealed six anomeric proton signals, which were observed at 5.45, 5.36, 5.01, 4.63, 4.49, and 4.48 ppm. In the HSQC spectra, these signals were correlated with carbon resonances at 93.7, 94.2, 106.3, 103.1, 101.2, and 101.7 ppm, respectively (Fig. 3). The ^31^P-NMR spectrum contained one signal for a phosphodiester group at −1.5 ppm. The six monosaccharides of E116 were designated as residues **A-F** in decreasing order of their proton chemical shift values. The ^1^H and ^13^C NMR chemical shifts for each sugar residue were assigned using a combination of 2D NMR experiments and by comparison with ^1^H and ^13^C NMR data that were previously published^14^,^15^ and are reported in Table 2. The TOCSY spectra showed cross-peaks of H1 with H2-H5 for residues **A**, **B**, and **D**, and cross-peaks of H1 with H2-H4 for residues **C**, **E**, and **F**. The assignment was completed by the following correlations in the COSY spectrum: H5/H6 for **A**, **B**, and **D**; and H4/H5 and H5/H6 for **C**, **E**, and **F**. The chemical shifts of the anomeric carbons of residues **A**, **B**, **D**, **E**, and **F** were characteristic for the pyranose ring form, whereas that of residue **C** was characteristic of a furanose ring form^16^. Residues **A**, **B**, and **D** were assigned to have the *gluco*-configuration, whereas residues **C**, **E**, and **F** have the *galacto*-configuration. The sequence of the monosaccharide residues within the repeating unit of the E116 polysaccharide was obtained by assignment of the inter-residue connectivities observed in the 2D NOESY and HMBC spectra (Table 2). The phosphate substitution position was revealed from ^1^H,^31^P-correlation experiments. The ^31^P resonance at *δ* −1.53 ppm showed connectivity to the H1 signal at *δ* 5.45 ppm (H-1 of residue **A**), the H6 signal at *δ* 3.97 ppm (H-6 of residue **D**), and the H5 signal at *δ* 3.62 ppm (H-5 of residue **D**). The final predicted structure of the hexasaccharide repeating unit of the *L*. *animalis/murinus* 116 polysaccharide, E116, is shown in Fig. 4.

Together, our results show that strains isolated from the intestines of healthy mice produce exopolysaccharides that are structurally identical across the IBD “−” strains, whereas strains isolated from intestine of mice with IBD produce exopolysaccharides whose chemical compositions differ greatly both between IBD “+”strains and with respect to IBD “−” strains.

### The lactobacilli and their exopolysaccharides trigger different *in vitro* responses in BM-DC

**Figure 5:**
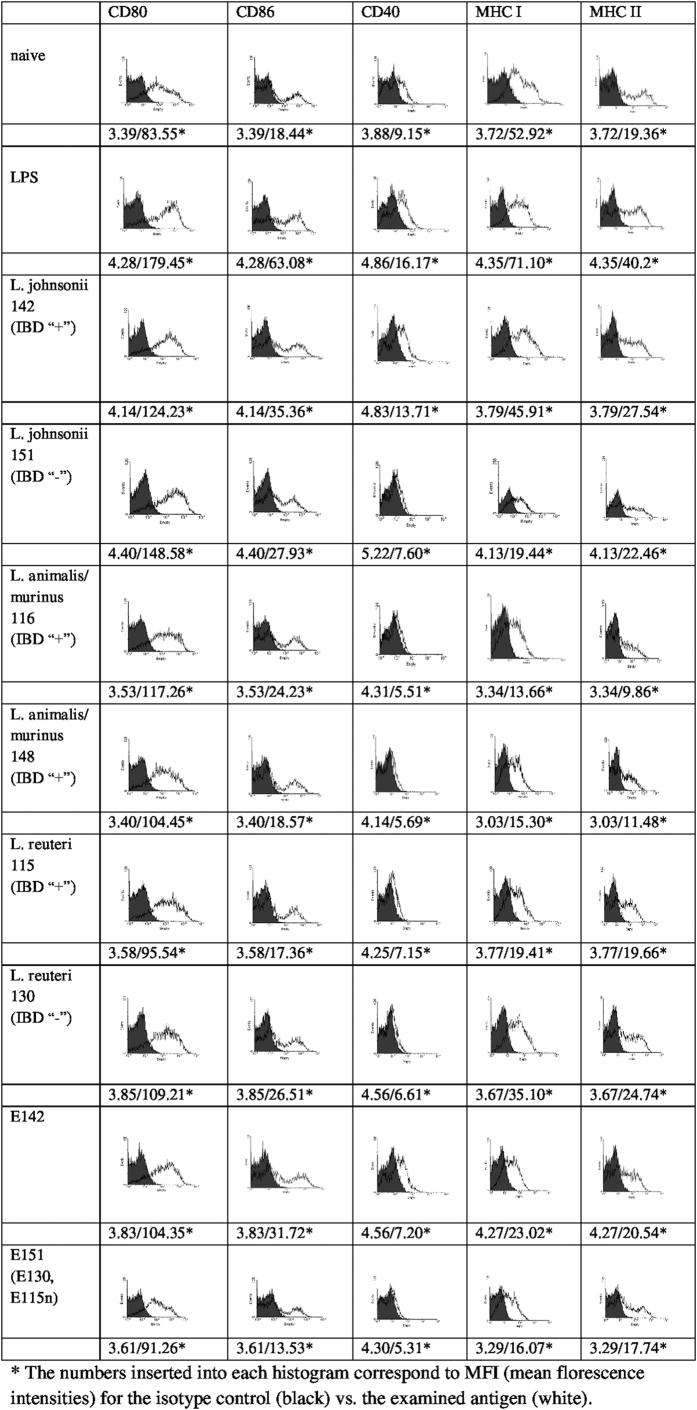
Flow cytometric analysis of DC differentiation marker expression on BM-DC activated with lactobacilli and their exopolysaccharides. The expressions of CD80 CD86, CD40, MHC I, and MHC II were estimated after 24 h of incubation with stimulators. The figures present results from one of three experiments.

When murine BM-DC were incubated with various lactobacillus strains or their exopolysaccharides, we observed that the capacities of the cells/polymers to up-regulate the expressions of MHC class I and II and co-stimulatory (CD80, CD86, CD40) molecules differed greatly based on the IBD status of the mice from which the bacteria were isolated. Cells were examined by flow cytometry, and the results are expressed as the mean fluorescence intensity (MFI). All strains were found to influence the expression of CD80. The effect of *L*. *johnsonii* 151 was the strongest (MFI 148.58), while that of *L*. *reuteri* 115 was weakest (MFI 95.54). *L*. *animalis/murinus* 148 and 116, *L*. *reuteri* 130 and 115, and *L*. *johnsonii* 151 induced minimal up-regulations of CD40, MHC I, and MHC II compared to untreated DC, whereas strong up-regulation was observed for *L*. *johnsonii* 142 (Fig. 5). Among the studied exopolysaccharides, only E142 significantly increased the differentiation of BM-DC (CD80, MFI 104.35; CD86, MFI 31.72), while E151 exhibited a tendency to induce the expression of CD80 (MFI 91.26) (Fig. 5). When BM-DC were stimulated with the other tested exopolysaccharides, the expression levels of the examined markers were similar to or lower than those in untreated controls (data not shown).

**Figure 6:**
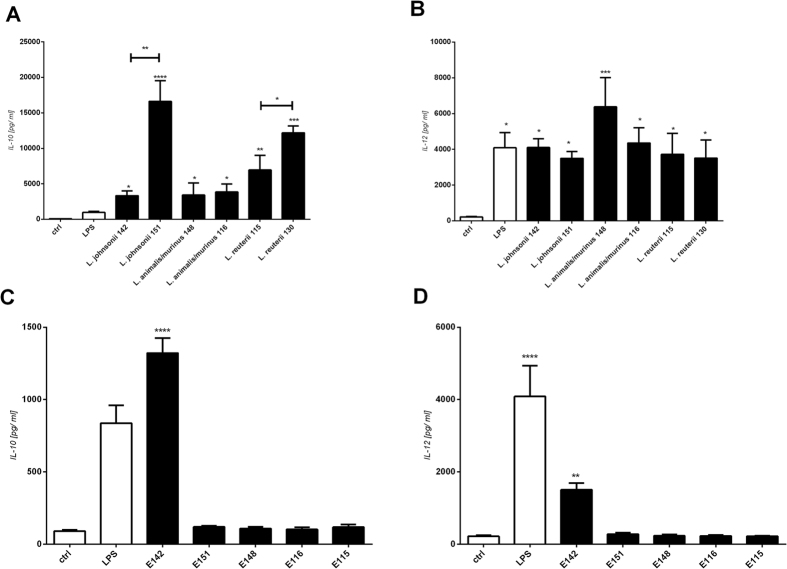
Cytokine production induced by *L*. *johnsonii* 142, *L*. *johnsonii* 151, *L*. *animalis*/*murinus* 148, *L*. *animalis/murinus* 116, *L*. *reuteri* 115, and *L*. *reuteri* 130, and by the structurally distinct exopolysaccharides, E142, E151, E148, E116, and E115. BM-DC from naive C56BL/6 mice were cultured with medium alone (ctrl), pure lipopolysaccharide from *E. coli* (LPS, 1 μg /ml), 10^7^ CFU/ml of lyophilized bacteria, or 10 μg/ml of exopolysaccharide. The levels of IL-10 (**A,C**) and IL-12p70 (**B,D**) in culture supernatants were determined by ELISA. Pooled results from four independent experiments are shown as mean ± SEM are shown; *p < 0.05, **p < 0.01, ***p < 0.001, and ****p < 0.0001.

BM-DC co-cultured for 24 h with lactobacilli or their exopolysaccharides were found to differ in their productions of IL-10 and IL-12p70 (Fig. 6A and B). Both IBD “+” and IBD “−” strains and their exopolysaccharides influenced the productions of both cytokines. Most importantly, the *L*. *johnsonii* 151 and *L*. *reuteri* 130 strains (IBD “−”) induced significantly higher levels of IL-10 than strains isolated from mice with IBD (*L*. *johnsonii* 142, *L*. *animalis/murinus* 148 and 116 and *L*. *reuteri* 115). In contrast, the bacterial strains from mice with IBD tended to induce higher levels of IL-12 than the strains from healthy mice. BM-DC stimulated with purified exopolysaccharides showed minimal cytokine production, with the exception of those treated with E142 (Fig. 6C and D). Cultivation of BM-DC with E142 induced the productions of IL-10 and IL-12p70, although to a lower degree than observed when whole *L*. *johnsonii* 142 cells were used.

In addition to testing responses in BM-DC, we profiled the T-helper (Th) response to *Lactobacillus* strains and their exopolysaccharides. We calculated the ratio of IL-10/IL-12, which is relevant to Th2 differentiation (Table 3). Although no statistically significant difference was detected, the strains from healthy mice and polymer E142 yielded the highest IL-10/IL-12 ratios, suggesting that these cells/polymer preferentially induced Th2-type responses.

### Responses of human Mo-DC to lactobacilli and their exopolysaccharides

**Figure 7:**
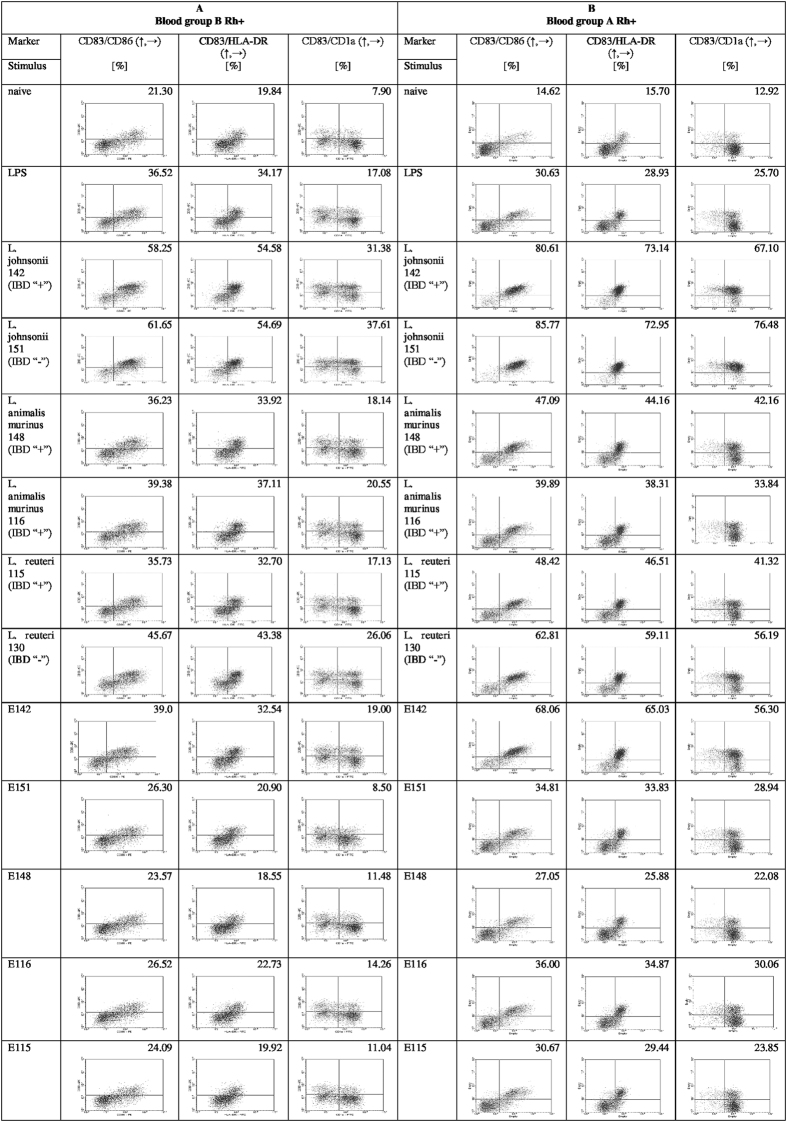
Flow cytometric analysis of DC differentiation marker expression on Mo-DC activated with lactobacilli and exopolysaccharide polymers. The expressions of CD83, CD86, CD1a, and HLA-DR were estimated after 24 h of incubation with stimulators. The presented results are from cells obtained from 6 to 10 blood donors of types B Rh+ (panel A) and A Rh+ (panel B).

The capabilities of the lactobacillus strains and their EPS to stimulate the differentiation of Mo-DC are summarized in Fig. 7. The tested Mo-DC markers were enhanced by cells obtained from A Rh+ donors (Fig. 7 panel B) but not B Rh+ donors (Fig. 7 panel A). Overall, the tested lactobacillus strains all stimulated differentiation of the human mononuclear cells, compared to non-stimulated or LPS-stimulated cells. It is worth noting that the two strains of *L*. *johnsonii* (142 and 151) induced the highest levels of marker expression among the studied stimuli. Among the exopolysaccharides, only E142 increased the stimulation index for Mo-DC compared to controls; in Mo-DC stimulated with the other exopolysaccharides, the levels of HLA-DR, CD1a, and maturation markers were similar to those in untreated controls.

**Figure 8:**
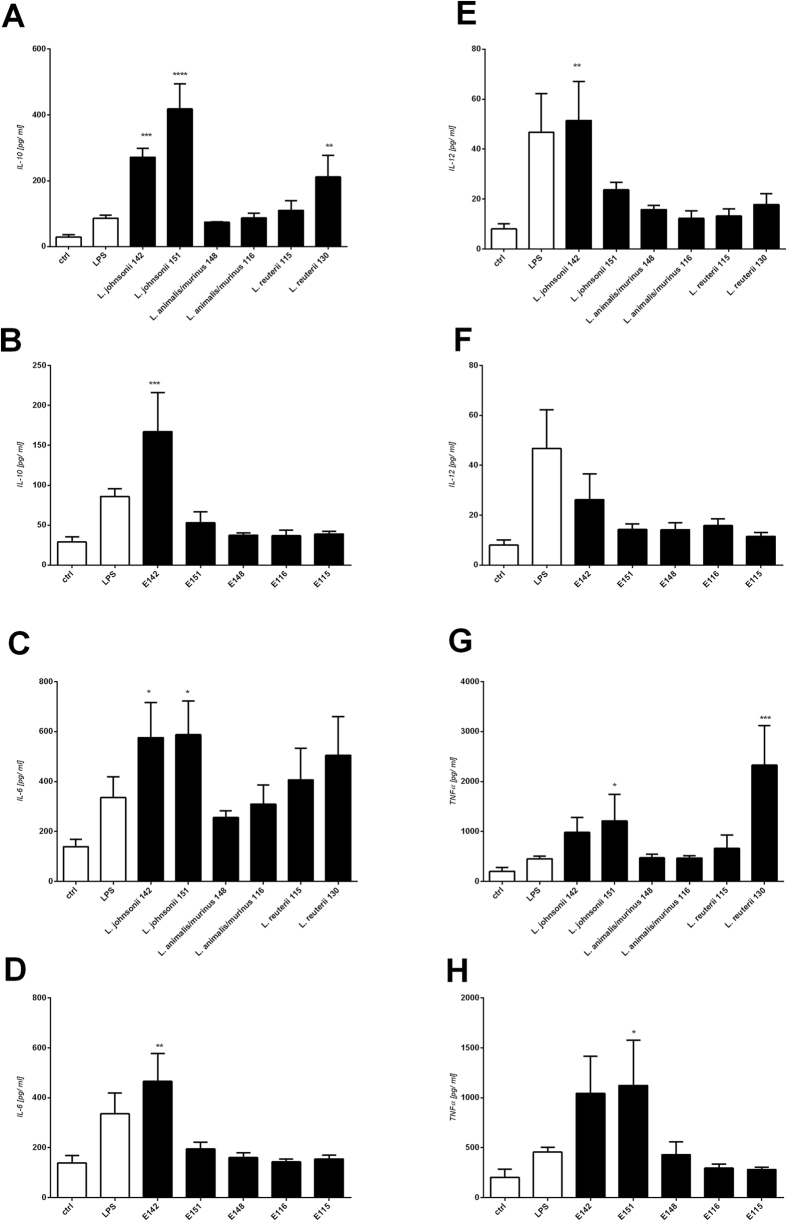
Cytokine production induced by *L*. *johnsonii* 142, *L*. *johnsonii* 151, *L*. *animalis/murinus* 148, *L*. *animalis/murinus* 116, *L*. *reuteri* 115, and *L*. *reuteri* 130 and the structurally distinct exopolysaccharides, E142, E151, E148, E116, and E115. Mo-DC isolated from human PBMC of 10 donors were purified and cultured with medium alone (ctrl), pure lipopolysaccharide from E. coli (LPS, 1 μg /ml), 10^7^ CFU/ml of lyophilized bacteria, or 10 μg/ml of exopolysaccharide. After 24 h, the levels of IL-10 (**A,B**), IL-6 (**C,D**), IL-12p70 (**E,F**), and TNF-α (**G,H**) in cell-free supernatants were measured by ELISA. Pooled results from five independent experiments are shown as the mean ± SEM; *p < 0.05; **p < 0.01, ***p < 0.001, and ****p < 0.0001.

In supernatants collected from Mo-DC cultured with lactobacilli or their exopolysaccharides, the cytokine production patterns appeared to be stimulus-dependent. In general, *L*. *johnsonii* (142 and 151), and *L*. *reuteri* 130 stimulated Mo-DC to produce large amounts of IL-10, with the highest up-regulation elicited by *L*. *johnsonii* 151 (Fig. 8A). *L*. *johnsonii* (142 and 151) also notably increased the level of IL-6 (p<0.05) (Fig. 8C). A significant level of IL-12 was detected after stimulation with *L*. *johnsonii* 142 (Fig. 8E), and *L*. *reuteri* 130 increased the level of TNF-α (Fig. 8G). There was no significant difference in the levels of IL-6, IL-10, IL-12, or TNF-α following stimulation with *L*. *animalis/murinus* 148, *L*. *animalis/murinus* 116, or *L*. *reuteri* 115, compared to untreated controls. Remarkably, most of the tested EPS polymers only weakly (if at all) induced cytokine secretion (Fig. 6B,D,F,H), indicating that they induced less of an immune response than the whole bacteria. The two exceptions were polymer E142, which stimulated the productions of IL-6 and IL-10 (Fig. 8B,D), and E151, which increased the level of TNF-α.

The observed differences in the productions of IL-12, IL-10, TNF-α, and IL-6 in response to stimulation by the different lactobacillus strains and exopolysaccharide polymers could help us understand which type of Th response is triggered in each case. Several cytokine ratios are known to be relevant for T cell differentiation; of them, we calculated the TNF-α/IL-10 and IL-10/IL-12 ratios to determine whether the Th cell differentiation tended toward Th1 or Th2 (Table 4). Interestingly, the strains isolated from healthy mice (*L*. *johnsonii* 151, *L*. *reuteri* 130) exhibited the highest IL-10/IL-12 ratio, corresponding to a Th2 profile, but their exopolysaccharide (E151 = E130) triggered a pro-Th1 profile. Mo-DC stimulated with *L*. *johnsonii* 142 or *L*. *reuteri* 115 (IBD “+”) also had elevated a high IL-10/IL-12 ratio. A marked elevation of IL-10/IL-12 but a high level of TNF-α/IL-10 ratio was observed for Mo-DC stimulated with *L*. *animalis/murinus* 116 and 148 (IBD “+”).

Among the exopolysaccharides, E142 expressed a pro-Th2 profile. There was no other statistically significant difference detected, although Mo-DC stimulated with E148 tended to exhibit an increased TNF-α/IL-10 ratio. Moreover, the immune responses elicited in Mo-DC treated with purified E142, E148, or E116 followed the same patterns as those caused by their respective EPS-producing strains.

## Discussion

IBD is a chronic and multifaceted relapsing inflammatory condition of the digestive tract. Clinical and experimental data suggest that IBD may result from alterations in the interactions between microorganisms and the intestinal immune system^17^,^18^. Specific host pathways linked to the microbial response in IBD include those responsible for sensing microbe-associated molecular patterns, activating T-cells and triggering their differentiation into T helper or T regulatory cells, up-regulating specific gut-homing receptors, and/or triggering Paneth cell function^19^,^20^,^21^. Still, little is known about the relationships linking IBD pathogenesis to individual microbes, their antigens, and the downstream immune alterations. Here, we compared the polysaccharide surface antigens of lactobacillus species isolated from mice with experimentally induced IBD and those obtained from healthy mice. We analyzed the chemical structures of the exopolysaccharides and compared their immunomodulatory properties with the immunomodulations caused by the corresponding whole bacteria. We previously determined the structures of EPS for *L*. *johnsonii* strain 142 (IBD “+”)^12^ and strain 151 (IBD “−”)^11^, and observed differences in their chemical compositions. To further expand upon these observations, we herein studied the EPS of *L*. *animalis/murinus* 148, *L*. *animalis/murinus* 116, *L*. *reuteri* 115 (IBD “+”), and *L*. *reuteri* 130 (IBD “−”). Interestingly, and consistent with our previous findings, we found distinct differences in EPS structures determined for IBD “+” versus IBD “−” species. Furthermore, we observed a high degree of diversity in the chemical compositions of the polymers from IBD “+” species, whereas those from IBD “−” species were structurally identical. *L*. *animalis/murinus* 148 and *L*. *animalis/murinus* 116, which produced identical polymers, each produced two different polymers distinguished by the lack of (E116, E148c) and the presence of (E116n, E148) acetylated amino sugars. Both polymers were negatively charged, but E116n (=E148) contained a phosphomonoester, whereas E116 (=E148c) contained a phosphodiester in its backbone chain. The structure of E116n (=E148) was previously reported^6^, while that of E116 (=E148c) is new. Polymer E116 (=E148c) is a branched heteropolysaccharide with a repeating unit that comprises six monosaccharide residues (three Gal and three Glc) and phosphorus. The presence of a phosphodiester bond between two glucose molecules in the main chain makes the structure uncommon. It is well known that phosphodiester bonds are central to life on Earth, as they make up the backbone of the nucleic acid strand. The walls of gram-positive bacteria may contain one or more secondary or accessory polymers that are attached via phosphodiester bonds to peptidoglycan (i.e., teichoic acid or teichuronic acids)^22^. Although similar phosphodiester bonds have been described for polysaccharides from pneumococci^23^ and meningococci^24^, to the best of our knowledge, no previous paper has described a phosphodiester bond within a polysaccharide of a *Lactobacillus*. *L*. *reuteri* 115 also produced two polymers differentiated by the lack of (E115n) or presence of (E115) acetylated amino sugars. Polymer E115n was found to be neutral, whereas E115 is negatively charged and contains a phosphomonoester in its backbone chain. The structure of E115n was characterized and found to be identical to those of E151 and E130 isolated from the IBD “−” species. In contrast, E115 was different and completely new.

After elucidating these differences in the structures of EPS from IBD “−” and IBD “+” species, we next evaluated the immunomodulatory effects of the bacterial cells and their respective EPS preparations on mouse murine BM-DC and human Mo-DC obtained from PBMC was examined. We found that the IBD “−” strains, *L*. *johnsonii* 151 and *L*. *reuteri* 130, induced significantly higher levels of IL-10 than strains isolated from mice with IBD (*L*. *johnsonii* 142, *L*. *animalis/murinus* 148 and 116, and *L*. *reuteri* 115) both in murine BM-DC and human Mo-DC. IL-10 is a cytokine of particular therapeutic interest in IBD. It has shown beneficial effects in experimental studies performed using IL-10 deficient mice^25^,^26^ and in clinical trials with IBD patients ingesting VSL#3 mixture^27^. Ulissse *et al*.^28^ reported that bacteria in the VSL#3 mixture can significantly increase the IL-10 level and reduce proinflammatory cytokine levels. In the present study, we did not observe any response to whole cells of IBD “−” species or the E151 polymer, suggesting that other antigens may be involved in stimulating IL-10 production by DC. Lammers *et al*.^29^ showed that genomic DNA from *Bifidobacterium* induced the secretion of IL-10 by PBMC. However, our group recently showed that although lactobacillus polysaccharides failed to change the DC maturation level or stimulate cytokine production, they could modulate the immune response patterns elicited by another bacterium^6^. Alternatively, they could exert *in vivo* effects by inducing regulatory DCs^30^. We found that *L*. *animalis/murinus* 148, *L*. *animalis/murinus* 116, and *L*. *reuteri* 115 (IBD “+”) and their corresponding exopolysaccharides, E148, E116, and E115, were immunologically silent. This is in line with the results of Lopez *et al*.^7^, who suggested that polysaccharide antigens surrounding bacterial cells may shield the bacteria from the immune cell response. In this way, EPS may play a rather indirect role in immune responses by protecting or shielding other surface molecules from their host receptors. In contrast, *L*. *johnsonii* 142 (IBD “+”) and its corresponding exopolysaccharide, E142, induced the differentiation of DC, triggered the production of cytokines, and preferentially induced the Th2-type response. Thus, the effect of lactobacilli appears to be strain dependent, and only exopolysaccharides with specific chemical structures can induce an immune response. It is well known that lactobacillus species vary phenotypically and functionally, and thus can invoke different mechanisms of action. Some strains induce immunostimulatory cytokines, whereas others are more effective at suppressing inflammatory responses^31^,^32^. For example, an increase in Th2 cytokines (IL-10 and IL-4) was observed in mice fed with *Lactobacillus delbrueckii* subspecies *bulgaricus* and *Lactobacillus casei*, whereas Th1 cytokines (IL-2 and IL-12) were induced in mice fed with *Lactobacillus acidophilus*^33^,^34^. Moreover, *Lactobacillus* species tend to reside on inflamed epithelial cells and may differentially modulate the production of cytokines, such as TNF-α or IL-10^35^,^36^. It is therefore important not only to determine what heterogeneous *Lactobacillus* populations inhabit inflamed and normal mucosa, but also to identify which specific bacteria can be used as a probiotic for IBD therapy.

Our results indicate that IBD “−” *Lactobacillus* species can be classified as anti-inflammatory, whereas the IBD “+” strains were immunologically silent with the exception of *L*. *johnsonii* 142. In addition to the distinct immunomodulations elicited by whole bacteria, we observed variations in the chemical structures of the exopolysaccharides expressed by these bacteria. This may indicate that, upon gut inflammation, such strains may switch to producing EPS with specific motifs that are absent from lactobacilli of the IBD “−” strains. Alternatively, inflamed intestinal mucosa could be inhabited by bacteria that harbor a particular EPS structure. Indeed, Iwamori *et al*.^37^ showed that administration of antibiotics can alter the glycolipids of both lactobacilli and host epithelial cells in the digestive tracts of mice. Furthermore, glycolipids with receptor activity for bacteria were metabolized in response to an alteration in the intestinal bacterial population. Other studies in rodents have indicated that the bacterial composition changes under colonic inflammation and/or infection^38^,^39^,^40^,^41^, suggesting that the inflamed mucosa and/or an altered inflammatory environment may selectively affect the growth and adherence of different bacterial species. Moreover, our group showed recently that there are differences in the immunoreactivities of sera from IBD patients versus healthy individuals. Interestingly, sera from IBD patients showed a low level of anti-polysaccharide antibodies and a high level of anti-glycolipids antibodies, whereas sera from healthy individuals showed much less reactivity to glycolipids than to polysaccharides^42^. These findings could indicate that the tolerance to the normal commensal flora of the intestine can break down during the inflammatory process. Recently, Alam *et al*.^43^ suggested that the microbiota can adapt to local environmental changes to form a determinable and temporary community. The microbiota must also stimulate pro-restitutive signaling and increase microbial migration and proliferation. This may suggest that, during co-evolution, host factors were able to influence the community structure and select for specific microorganisms with beneficial properties.

In conclusion, we herein present comparative studies of the bacteria of IBD “+” and IBD “−” mice and their surface antigens, and our results may shed light on the mechanisms underlying the pathogenesis of IBD. The study of these and other host-bacteria relationships during inflammation and disease recovery may be a fruitful source for mining and isolating microorganisms with therapeutic potential, such as for the probiotic therapy of this disease. This information may contribute to the development of functional and comparative approaches aimed at unraveling the functionality of these bacteria in the intestinal tract. Gaining new insights into the antigens and the molecular mechanisms they use to induce an effective immune response at mucosal sites could help us define immunotherapeutic approaches and prevent disease in the future.

## Materials and Methods

### Bacterial strains and isolation of exopolysaccharides

Strains of *Lactobacillus johnsonii* 142, *Lactobacillus animalis*/*murinus* 148, *Lactobacillus animalis*/*murinus* 116, and *Lactobacillus reuteri* 115 were isolated from the intestinal tracts of mice with experimentally induced inflammatory bowel disease (IBD “+”), whereas *Lactobacillus johnsonii* 151 and *Lactobacillus reuteri* 130 were isolated from healthy mice (IBD “−”). These bacterial species were a gift from M. Strus and P.B. Heczko (CM UJ Krakow, Poland). CD4+CD45RB high T cell transfer SCID mice with colitis were housed under SPF conditions and used as an animal model of IBD^44^. To identify bacteria, cell morphology was assessed and phenotypic identification was performed using commercial identification systems (API 20E, API20A, APIStaph, APIStrep, API50CHL, all from bioMerieux; and BBL Crystal ID System from BD, USA). The genus designation of *Lactobacillus* was established by PCR amplification of the 16S–23S rRNA^45^ or rep-PCR using GTG5 primers^46^. The strains were stored at −75 °C in MRS broth (deMan, Rogosa and Sharpe; Biocorp) supplemented with 20% glycerol.

Bacteria were cultivated in MRS broth under anaerobic conditions at 37 °C for 48 h. Cells were harvested by centrifugation at 6000 rpm (4 °C, 20 min) and washed twice with PBS and once with MilliQ water. The freeze-dried bacterial mass was extracted with 10% TCA (25 °C, 2.5 h) and then centrifuged at 14,500 g for 20 min. The pellet was discarded and the exopolysaccharides from the supernatant were precipitated with 5 volumes of cold 96% ethanol (4 °C, overnight), collected by centrifugation at 14,500 g (4 °C, 50 min), suspended in water, dialyzed for 48 h against water, and freeze-dried.

### Purification of exopolysaccharides

The exopolysaccharide antigens of lactobacilli were isolated and purified as previously described^12^. Briefly, the freeze-dried preparation of crude exopolysaccharide (EPS) antigens (20 mg) was dissolved in 1 ml of buffer (50 mM Tris-HCl, pH 7.5, 10 mM MgCl_2_) and treated with DNase (210 μg; Sigma) and RNase (210 μg; Sigma) at 37 °C for 6 h. This step was followed by hydrolysis with *Streptomyces griseus* protease (447 μg, 37 °C, overnight; Sigma). Finally, the preparation was dialyzed against water at 4 °C for 24 h. The exopolysaccharides were further purified by ion exchange chromatography on a DEAE-Sephadex A-25 column (1.6 × 20 cm; Pharmacia). The neutral fractions were eluted with 20 mM Tris buffer, pH 8.2, and the charged fractions were released with an NaCl gradient (0–2 M) in 20 mM Tris buffer, pH 8.2, at a flow rate of 0.5 ml/min. The fractionation was monitored at 220 nm with a UV-VIS absorbance detector (to detect contaminants; Pharmiacia) and a differential refractometer (Knauer), the carbohydrate content was analyzed by the phenol/sulfuric acid method^47^, and the phosphate content was determined as described previously^48^. The polysaccharide-containing fractions were pooled, desalted by dialysis against water at 4 °C for 24 h, lyophilized, and further purified by gel permeation chromatography on a Toyopearl HW-55S column (1.6 × 100 cm; Tosoh Bioscience LLC) fitted to an FPLC system (Amersham Pharmacia Biotech). The fractions were eluted with 0.1 M ammonium acetate buffer. The column effluents were monitored with an absorbance detector at λ = 280 nm (for protein contamination), with a differential refractometer (Knauer), and for the carbohydrate content (see above). The purified EPS were tested with a Limulus amebocyte lysate (LAL) assay (PyroGene™ Recombinant Factor C Endotoxin Detection Assay; Lonza). The levels of endotoxin were below 0.1 EU per 1 μg of pure exopolysaccharide.

### Sugar and methylation analysis and determination of the absolute configuration

The monosaccharides were identified by gas-liquid chromatography-mass spectrometry (GLC-MS) of alditol acetates. Each sample was prepared by hydrolysis (10 M HCl at 80 °C for 25 min) followed by reduction (alkaline, pH 8, NaBH_4_ solution for 16 h in the dark) and peracetylation [acetic anhydride and methylimidazole (1:0.2, v/v) at 25 °C for 15 min]^49^. Standards of arabinose, mannose, rhamnose, fucose, glucose, galactose, glucosamine, galactosamine, mannosamine, and xylose were prepared for comparison. The system used was a Hewlett-Packard (HP) 5971 A instrument equipped with an HP-1 capillary column, and the applied temperature gradient was 150 to 270 °C at 8 °C/min. For methylation analysis, the exopolysaccharide samples were first peracetylated with a mixture of trifluoroacetic anhydride and acetic acid (2:1 vol/vol, 25 °C, 10 min)^50^ and then permethylated according to the method of Ciukanu & Kerek^51^. The reaction product was extracted with a water-chloroform mixture. The methylated polysaccharide was hydrolyzed (2 M TFA at 120 °C for 2 h), reduced with NaBD_4_, and acetylated using the conditions described above for the sugar analysis. The acetylated (*S*)-2-butanol glycoside derivative was subjected to butanolysis [300 μl (S)-2-butanol and 20 μl AcCl, 100 °C, 3 h], and the absolute configuration was determined by GLC-MS. The resulting mixture was trimethylsilylated (1:3:9 trimethylchlorosilane:hexamethyldisilazane:pyridine, room temperature, 30 min)^52^ and then analyzed by GLC-MS as described for the sugar analysis. Peak retention times and mass spectra analysis were used to identify sugar derivatives.

### NMR spectroscopy

The NMR spectra were obtained on a Bruker 600 MHz Avance II spectrometer using a 5 mm ^1^H/^13^C/^15^N/^31^P inverse detection probe equipped with a z-gradient. The NMR spectra were obtained for ^2^H_2_O solutions of the exopolysaccharides at 25 °C using acetone (δ_H_ 2.225, δ_C_ 31.05) as an internal reference. The exopolysaccharides (10 mg) were repeatedly exchanged with ^2^H_2_O with intermediate lyophilization. The data were acquired and processed using standard Bruker software. The processed spectra were assigned with help from the SPARKY NMR analysis program^53^. The signals were assigned from one- and two-dimensional experiments (COSY, TOCSY, NOESY, HSQC with and without carbon decoupling, HSQC-TOCSY, and HMBC) from the Bruker pulse sequence library. TOCSY experiments were carried out with mixing times of 30, 60, and 100 ms. The delay time in HMBC was 60 ms and the mixing time for NOESY was 200 ms. The HSQC-TOCSY experiment was carried out with a TOCSY mixing time of 80 ms.

### Preparation and activation of murine bone marrow-derived dendritic cells

The C57BL/6 mouse bone marrow-derived dendritic cells (BM-DC) were isolated from femurs and tibias, washed, and cultured in RPMI 1640 (Gibco) supplemented with 100 U/ml penicillin, 100 U/ml streptomycin, 2 mM L-glutamine, 1 mM sodium pyruvate, 50 μM 2-mercaptoethanol, and 10% (v/v) fetal bovine serum (FBS, HyClone) containing 40 ng/ml of recombinant murine GM-CSF (Strathman) and 10 ng/ml of recombinant murine IL-4 (Strathman). Clusters of growing BM-DC remained loosely attached to the tissue culture vessel. Their DC phenotypes were confirmed after 6 days of culture. At that time, the BM-DC were transferred to 24-well plates (1.0 × 10^6^ cells/ml), incubated in culture medium supplemented with GM-CSF for 24 h, and then incubated for 24 h with or without (+/−):

1. 10 μg/ml of EPS antigen: a) exopolysaccharide E142 isolated from *L*. *johnsonii* 142 (E142), b) exopolysaccharide E151 (structurally identical to E130 and E115n) isolated from *L*. *johnsonii* 151 (E151), c) exopolysaccharide E148 (structurally identical to E116n) isolated from *L*. *animalis*/*murinus* 148 (E148), d) exopolysaccharide E116 (structurally identical to E148c) isolated from *L*. *animalis*/*murinus* 116 (E116), or f) exopolysaccharide E115 isolated from *L. reuteri* 115 (E115);
2. 10^7^ CFU/ml of lyophilized lactobacilli: *L*. *johnsonii* 142, *L*. *johnsonii* 151, *L*. *animalis/murinus* 116, *L*. *animalis/murinus* 148, or *L*. *reuteri* 115; or
3. 5 μg of LPS from *E*. *coli* C600.

The levels of IL-10 and IL-12p70 in culture supernatants were determined using Mouse BD OptEIA^TM^ ELISA kits (BD Bioscience Pharmingen, USA) according to the manufacturer’s instructions. The experiments were approved by the 1st Local Committee for Experiments with the Use of Laboratory Animals, Wroclaw, Poland (no. 15/2009).

### Preparation and activation of human peripheral blood mononuclear cells

The human peripheral blood mononuclear cells (PBMC) were obtained from buffy-coats of 6–10 healthy volunteers (B Rh+ or A Rh+ blood group) from the Military Blood Donors Centre in Wroclaw (Poland) using Gradisol G (Aqua-Med, Poland) gradient centrifugation for 30 min at 1000 × g. Monocytes were purified using a MACS CD14 isolation kit (Miltenyi Biotec, Bergisch Gladbach, Germany). Flow cytometry was used to confirm the purity of isolated cells. The procedure allowed for concentration of CD14+ cells to approx. 80%. The cells were distributed to six-well tissue culture plates (0.5–1.5 × 10^6^ cells/ml) in fresh RPMI 1640 (Gibco) supplemented with 100 U/ml penicillin, 100 U/ml streptomycin, 2 mM L-glutamine, 1 mM sodium pyruvate, 50 μM 2-mercaptoethanol, and 10% (v/v) fetal bovine serum (FBS, HyClone) containing 100 ng/ml of recombinant murine GM-CSF (Strathman) and 50 ng/ml of recombinant murine IL-4 (Strathman), at 37 °C in a humidified atmosphere supplemented with 5% CO_2_. To generate standard mononuclear dendritic cells (Mo-DC), cells were cultured for 6 days. The purity of the Mo-DC was assessed by flow cytometric analysis of CD11c expression, and was found to be 70–75% (data not shown). For experiments, cells (0.5 × 10^6^ cells/ml) were incubated for 24 h with or without (+/−):

1. 10 μg/ml of EPS antigen: a) exopolysaccharide E142 isolated from *L*. *johnsonii* 142 (E142), b) exopolysaccharide E151 (structurally identical to E130 and E115n) isolated from *L*. *johnsonii* 151 (E151), c) exopolysaccharide E148 (structurally identical to E116n) isolated from *L*. *animalis*/*murinus* 148 (E148), d) exopolysaccharide E116 (structurally identical to E148c) isolated from *L*. *animalis*/*murinus* 116 (E116), or f) exopolysaccharide E115 isolated from *L. reuteri* 115 (E115);
2. 10^7^ CFU/ml of lyophilized lactobacilli: *L*. *johnsonii* 142, *L*. *johnsonii* 151, *L*. *animalis/murinus* 116, *L*. *animalis/murinus* 148, or *L*. *reuteri* 115; or
3. 5 μg of LPS from *E*. *coli* C600.

The levels of IL-6, IL-10, TNF-α, and IL-12p70 in culture supernatants were determined by using Human BD OptEIA^TM^ ELISA kits (BD Bioscience Pharmingen, USA) according to the manufacturer’s instructions. To minimize interindividual variation, all experiments were performed with cells obtained from 6 to 10 blood donors of the same blood group (A Rh+ or B Rh+). Samples were obtained with patients’ written informed consent. The study protocol was approved by the Medical Ethics Committee of Wroclaw Medical University (Wroclaw, Poland; no. 100/2009) and the study was conducted in accordance with the Helsinki Declaration of 1975, as revised in 1983.

### Flow cytometric analysis

For direct immunofluorescence staining of murine DC, the following phycoerythrin (PE)-conjugated rat anti-mouse antibodies were used (BD Biosciences Pharmingen): CD11c-PE (dilution 1:100), CD80-PE (dilution 1:600), CD86-PE (dilution 1:600), and CD40-PE (dilution 1:200). For direct cell staining, 50 μl of cell suspension was mixed with 50 μl of diluted antibody, and the mixture was incubated at 4 °C for 45 min. For indirect cell staining, 50 μl of cell suspension was incubated with an equal volume of unconjugated mouse anti-mouse I-A and MHC II (dilutions for both, 1:100) antibodies for 30 min at 4 °C, washed, and further incubated at 4 °C for 45 min with AlexaFluor 488-conjugated rat anti-mouse antibody (dilution 1:400; BD Biosciences Pharmingen).

Human dendritic cells were labeled directly with monoclonal antibodies against CD11c-APC (dilution 1:100), HLA-DR-FITC (dilution 1:50), CD83-APC (dilution 1:50), CD86-PE (dilution 1:100), CD1a-FITC (dilution 1:50) (BD Biosciences Pharmingen). For cell staining, 50 μl of cell suspension was mixed with 50 μl of diluted antibody, and the mixture was incubated at 4 °C for 45 min. Appropriate isotype antibodies were used as controls to determine non-specific binding. Cells were analyzed using a FACSCalibur flow cytometer (Becton-Dickinson, USA).

### Statistical analysis

Data are expressed as the mean ± SEM. Statistical analysis was performed by one-way analysis of variance (ANOVA) followed by Sidak’s multiple comparison test. Analyses were performed using the GraphPad Prism 5.04 software (GraphPad, San Diego, CA USA). A p value < 0.05 was considered significant.

## Additional Information

**How to cite this article**: Górska, S. *et al*. Structural and immunomodulatory differences among lactobacilli exopolysaccharides isolated from intestines of mice with experimentally induced inflammatory bowel disease. *Sci. Rep*. **6**, 37613; doi: 10.1038/srep37613 (2016).

**Publisher’s note**: Springer Nature remains neutral with regard to jurisdictional claims in published maps and institutional affiliations.

## Figures and Tables

**Table 1:** ^1^H, ^13^C and ^31^P NMR chemical shifts (ppm) and selected inter-residue connectivities from the anomeric protons of E115 from *Lactobacillus reuteri* strain 115.

	Sugar residue	Chemical shift (ppm) for							
		H1	H2	H3	H4	H5	H6 H6‘	CH_3_CO	Selected connectivities (ppm)
		C1	C2	C3	C4	C5	C6		
A	→2,3)-α-D-Glc*p*-(1→	5.71	3.87	3.96	3.58	3.71	3.79		**AH1-DH3 (3.87)**
		96.1	73.4	78.6	68.6	72.9	61.8		**AH1-DH1 (4.81)**
B	→3)-α-D-Gal*p*-(1→	5.27	3.97	3.93	3.29	4.15	3.80		**BH1-AH2 (3.87)**
		95.7	67.7	81.3	69.6	71.3	61.8		**BH1-AC2 (82.5)**
									**BH1-AH5 (3.71)**
C	P→6)-β-D-Gal*f*-(1→	5.26	4.18	4.14	4.14	4.18	4.22 4.14		**CH1-AH3 (3.96)**
		108.8	82.5	78.0	83.3	71.4	68.2		**CH1-CC2 (82.5)**
									
D	→3)-β-D-Glc*p*NA*c*-(1→	4.81	3.94	3.87	3.73	3.53	3.76 3.98	1.93	**DH1-GH4 (4.32)**
		102.8	54.9	79.8	71.6	75.9	61.6	22.1	**DC1–GC4 (76.1)**
E	→6)-β-D-Glc*p*NA*c*-(1→	4.71	3.81	3.62	3.92	3.64	4.27 3.93	1.95	**EH1 – BH3 (3.93)**
		103.8	56.4	75.6	70.7	74.8	69.2	22.3	**EC1-BC3 (81.3)**
F	→3)-β-D-Glc*p*-(1→	4.56	3.42	3.71	3.64	3.63	4.03 3.86		**FH1-EH6 (3.93)**
		103.5	73.4	79.8	71.8	75.4	61.0		**FC1–EC6 (69.2)**
G	→4)-β-D-Gal*p*-(1→	4.54	3.53	4.24	4.32	3.81	3.81 3.80		**GH1- FH3 (3.71)**
		103.6	71.4	77.8	76.1	75.0	61.5		**GC1–FC3 (79.8)**
P		0.53							**P – CH6, H6’ (4.22, 4.14)**

Spectra were obtained for ^2^H_2_O solutions at 25 °C. Acetone (δ_H_ 2.225, δ_C_ 31.05 ppm) was used as an internal reference.

**Table 2:** ^1^H, ^13^C and ^31^P NMR chemical shifts (ppm) and selected inter-residue connectivities from the anomeric protons of E116 from *Lactobacillus animalis/murinus* strain 116.

	Sugar residue	Chemical shift (ppm) for						
		H1	H2	H3	H4	H5	H6 H6’	Selected inter-residue connectivities (ppm)
		C1	C2	C3	C4	C5	C6	
A	→4)-α-D-Glc*p*-(1→P	5.45	3.60	3.83	3.76	4.05	3.81 3.96	**AH1-DH6 (4.21)**
		93.7	68.4	69.9	76.8	69.1	58.9	
B	→2)-α-D-Glc*p*-(1→	5.36	3.70	3.90	3.49	3.94	3.80	**BH1-FH3 (3,75)**
		94.2	79.9	70.2	68.0	70.2	59.8	**BH1-FH4 (4.26)**
								**BH1-BC5 (70.2)**
C	β-D-Gal*f*-(1→	5.01	4.08	4.04	3.97	3.79	3.69	**CH1-EH6 (4.02, 3.87)**
		106.3	79.7	75.2	80.8	69.4	61.5	**CC1-EC6 (64.6)**
D	P→6)-β-D-Glc*p*-(1→	4.63	3.37	3.48	3,40	3,62	3.97 4.21	**DH1-BH2 (3.70)**
		103.1	72.0	74.1	68.3	73.5	63.7	**DC1–BC2 (79.9)**
E	→3,6)-β-D-Gal*p*-(1→	4.49	3.36	3.66	3.59	3.62	4.02 3.87	**EH1 – AH4 (3.76)**
		101.2	71.5	77.4	73.7	73.6	64.6	**EC1-AC4 (76.8)**
F	→3)-β-D-Gal*p*-(1→	4.48	3.62	3.75	4.26	3.75	3.80 3.96	**FH1-EH3 (3.66)**
		101.7	69.7	76.8	63.7	73.7	58.8	**FC1–EC3 (77.4)**
P		−1.5						**P – AH1 (5.45)**
								**P – DH6 (3.97)**
								**P – DH5 (3.62)**

Spectra were obtained for ^2^H_2_O solutions at 25 °C. Acetone (δ_H_ 2.225, δ_C_ 31.05 ppm) was used as an internal reference.

**Table 3:** Ratio of IL-10/IL-12 cytokines secreted by BM-DC stimulated with selected bacteria and exopolysaccharides.

Stimulus	IL-10/IL-12 ratio
*L*. *johnsonii* 142 (IBD “+”)	0.9
*L*. *johnsonii* 151 (IBD “−”)	1.9
*L. animalis/murinus* 148 (IBD “+”)	1.4
*L. animalis/murinus* 116 (IBD “+”)	1.5
*L. reuteri* 115 (IBD “+”)	1.1
*L. reuteri* 130 (IBD “−”)	3.4
E142 (IBD “+”)	0.9
E151 (IBD “−”)	0.4
E148 (IBD “+”)	0.4
E116 (IBD “+”)	0.4
E115 (IBD “+”)	0.5

**Table 4:** Ratio of TNF-α/IL-10 (pro-Th1), IL-10/IL-12 (pro-Th2) cytokines secreted by human Mo-DC stimulated with selected bacteria and exopolysaccharides.

Stimulus	IL-10/IL-12 ratio	TNF-α/IL-10 ratio
*L*. *johnsonii* 142 (IBD “+”)	8.4	8.0
*L*. *johnsonii* 151 (IBD “−”)	11.7	6.4
*L. animalis/murinus* 148 (IBD “+”)	3.7	7.1
*L. animalis/murinus* 116 (IBD “+”)	5.4	7.1
*L. reuteri* 115 (IBD “+”)	8.3	5.4
*L. reuteri* 130 (IBD “−”)	10.0	9.2
E142 (IBD “+”)	7.3	6.5
E151 (IBD “−”)	4.5	15.6
E148 (IBD “+”)	3.4	10.0
E116 (IBD “+”)	2.7	7.8
E115 (IBD “+”)	3.6	5.8
